# Advancing data reuse in phyloinformatics using an ontology-driven Semantic Web approach

**DOI:** 10.1186/1755-8794-6-S3-S5

**Published:** 2013-11-11

**Authors:** Maryam Panahiazar, Amit P Sheth, Ajith Ranabahu, Rutger A Vos, Jim Leebens-Mack

**Affiliations:** 1 Ohio Center for Excellence in Knowledge-enabled Computing (kno.e.sis) College of Computer Science and Engineering, Wright State University, Dayton, OH, USA; 2 Department of Plant Biology, University of Georgia, Athens, GA, USA; 3 Naturalis Biodiversity Center, Leiden, the Netherlands; 4 Bioinformatics Institute, University of Georgia, Athens, GA, USA

## Abstract

Phylogenetic analyses can resolve historical relationships among genes, organisms or higher taxa. Understanding such relationships can elucidate a wide range of biological phenomena, including, for example, the importance of gene and genome duplications in the evolution of gene function, the role of adaptation as a driver of diversification, or the evolutionary consequences of biogeographic shifts. Phyloinformaticists are developing data standards, databases and communication protocols (e.g. Application Programming Interfaces, APIs) to extend the accessibility of gene trees, species trees, and the metadata necessary to interpret these trees, thus enabling researchers across the life sciences to reuse phylogenetic knowledge. Specifically, Semantic Web technologies are being developed to make phylogenetic knowledge interpretable by web agents, thereby enabling intelligently automated, high-throughput reuse of results generated by phylogenetic research. This manuscript describes an ontology-driven, semantic problem-solving environment for phylogenetic analyses and introduces artefacts that can promote phyloinformatic efforts to promote accessibility of trees and underlying metadata. *PhylOnt *is an extensible ontology with concepts describing tree types and tree building methodologies including estimation methods, models and programs. In addition we present the *PhylAnt *platform for annotating scientific articles and NeXML files with *PhylOnt *concepts. The novelty of this work is the annotation of NeXML files and phylogenetic related documents with PhylOnt Ontology. This approach advances data reuse in phyloinformatics.

## Background

Forty years ago, Theodosius Dobzhansky asserted "Nothing in biology makes sense except in the light of evolution" [1], and phylogenetic trees offer a historical representation of the evolutionary process. Since Darwin and Haeckel published their iconic tree figures some 150 years ago [2,3] phylogenies have provided the historical framework for elucidating the evolution of form and function [4]. In addition to estimating organismal relationships and the timing of gene duplications [5,6], phylogenies can be applied to many more research questions. For example, they can be used to inform prediction of protein function [7] and investigations of disease transmission [8]. More generally, phylogenies provide a unifying context across the life sciences for investigating the diversification of biological form and function from genotype to phenotype.

The increased interest in using and reusing phylogenies has exposed major limitations in the accessibility and reusability of published phylogenetic trees and the data used to estimate these trees. Most published phylogenetic trees can only be found in text and graphical format embedded in printed or electronic research publications [9,10]. As a consequence, these trees are typically inaccessible for semantic processes, including web-based identification and acquisition of trees, analytical methods, or the data on which phylogenetic inferences are based. This greatly limits the ability of biologists to reuse gene and species trees in meta-analyses with other structured sources.

There is a wealth of information that surrounds each phylogenetic study, including comparative data such as morphological character state matrices and nucleotide or amino acid sequence alignments, methodological descriptions such as substitution model and provenance information. All of this information is represented in a variety of different formats ranging from unstructured data such as texts and images in published technical reports and academic articles to semi-structured data such as tables and key delimited records and structured data such as database entries, and XML files. This variation of formats poses informatics challenges to the integration of diverse data and the generation of federated queries to answer specific research questions.

Here we present results to promote an ontology-driven, semantic problem-solving solution for phylogenetic analyses and downstream use of phylogenetic trees. We have constructed a network of concepts and defined them in an ontology, *PhylOnt*, and provide examples for how these concepts can be used to annotate publications and data files. *PhylOnt *is an extensible ontology that describes the methods employed to estimate trees given a data matrix, models and programs used for phylogenetic analysis and descriptions of phylogenetic trees as well as provenance information.

The common vocabulary included in *PhylOnt *will facilitate the integration of heterogeneous data types derived from both structured and unstructured data sources. Annotation tools for tagging *PhylOnt *terms in scientific literature and NeXML formatted data files are also presented. NeXML is an exchange standard for representing taxa, phylogenetic trees, character matrices (e.g. sequence alignments) and associated metadata [11]. As such, well annotated NeXML files could contain the minimum information about a phylogenetic analysis (MIAPA) [4] necessary to enable reproducibility and reuse of phylogenetic inferences.

In addition, we evaluate *PhylOnt *using formal metric-based and annotation-based approaches. This assessment indicates that more than half of the connections between *PhylOnt *classes are information-rich. Further, an analysis of exemplar publications indicates that for phylogenetic operations, methods, models and programs the majority of phylogenetic concepts can be accurately annotated using *PhylOnt*.

### Related work

The work described here builds on the needs assessment described by Stoltzfus et al [10] and our research previously presented at the IEEE ICSC 2011 [12], iEvoBio 2011 [13], the W3C Workshop on Data and Services Integration [14], Translational Medicine Conference at AMIA 2012 [15,16] and IEEE International Conference on Bioinformatics and Biomedicine [17]. Recent "PhyloTastic" hackathons [18] have also developed resources to promote the reuse of published trees and underlying metadata.

Other prior art with regard to the Semantic Web-ready definition of phylogeny-related concepts exists in the form of previously published ontologies, most notably the Comparative Data Analysis Ontology (CDAO) [19] and the Embrace Data And Methods (EDAM) ontology [20]. CDAO is an ontology that describes fundamental data and transformations commonly found in the domain of evolutionary analyses. CDAO [19] includes concepts relevant to phylogenies such as nodes, edges, branches, and networks, but concepts relating to phylogenetic analysis methods or provenance are omitted. EDAM [20] is an ontology developed for general bioinformatics concepts including operations, topics, types and formats. EDAM includes phylogeny-related concepts but phylogenetic analysis terms relating to methods, models and programs are either not reported in EDAM or have not been explicitly defined under a correct hierarchy for phylogenetic analysis purposes. *PhylOnt *aims to cover the general concepts necessary to describe phylogenetic analyses. These ontologies are explained and compared with *PhylOnt *in [17].

## Methods

*PhylOnt *aims to characterize selected "phylogenetic resource" concepts and the relationships among these concepts. In this context, we define a "phylogenetic resource" as any uniquely identifiable object or procedure from the domain of phylogenetic research, ranging from the granular, e.g. a specific node in a tree, to the holistic, e.g. a study, or a step in an analysis workflow. *PhylOnt *includes concepts for estimation programs, models of evolution, methods of analysis, search algorithms, support assessments, and relevant provenance information. *PhylOnt *will grow as new tree estimation technologies are developed and used in published phylogenetic studies. Developing an ontology and using it to annotate the data and services in analysis workflows can provide a foundation for other semantic technologies, such as concept-based searches and comprehensive federated queries over data sources.

### Systematic approach for ontology development

**Figure 1:**
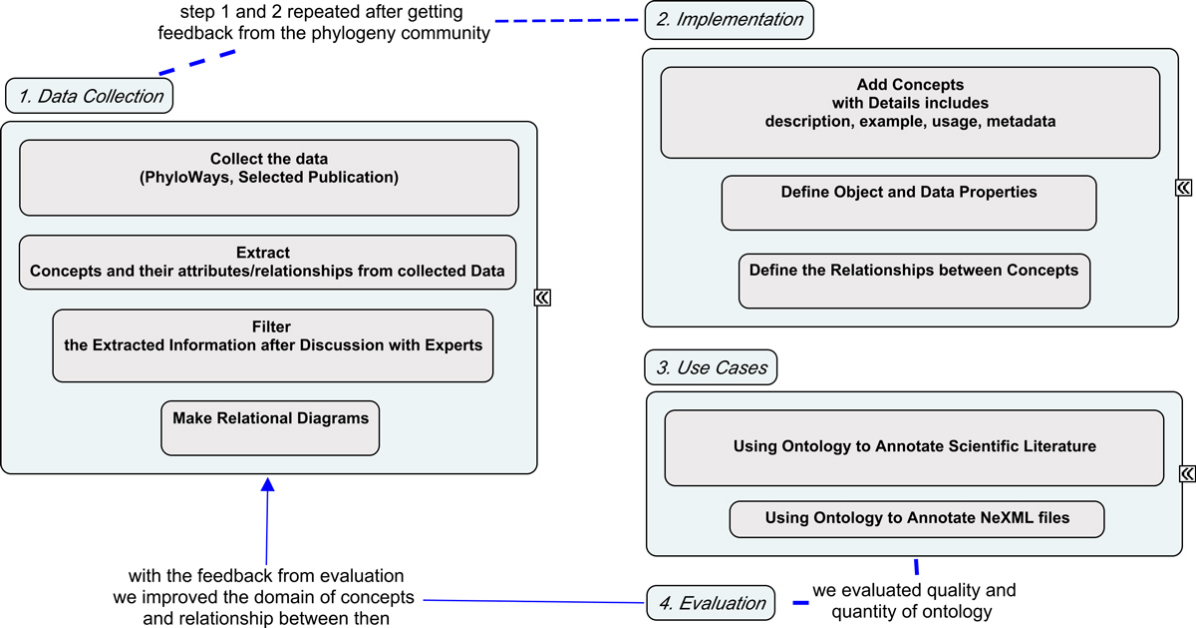
**Systematic approach for ontology development**. Our phylogenetics ontology development efforts started with data collection with experts in the field, making analytical diagram for the key concepts such as methods, models and programs. The ontology was formally constructed using Protege. Use cases were employed to evaluate the benefit of *PhylOnt *concepts for annotation of published phylogenetic trees, their estimation and underlying data.

**Figure 2:**
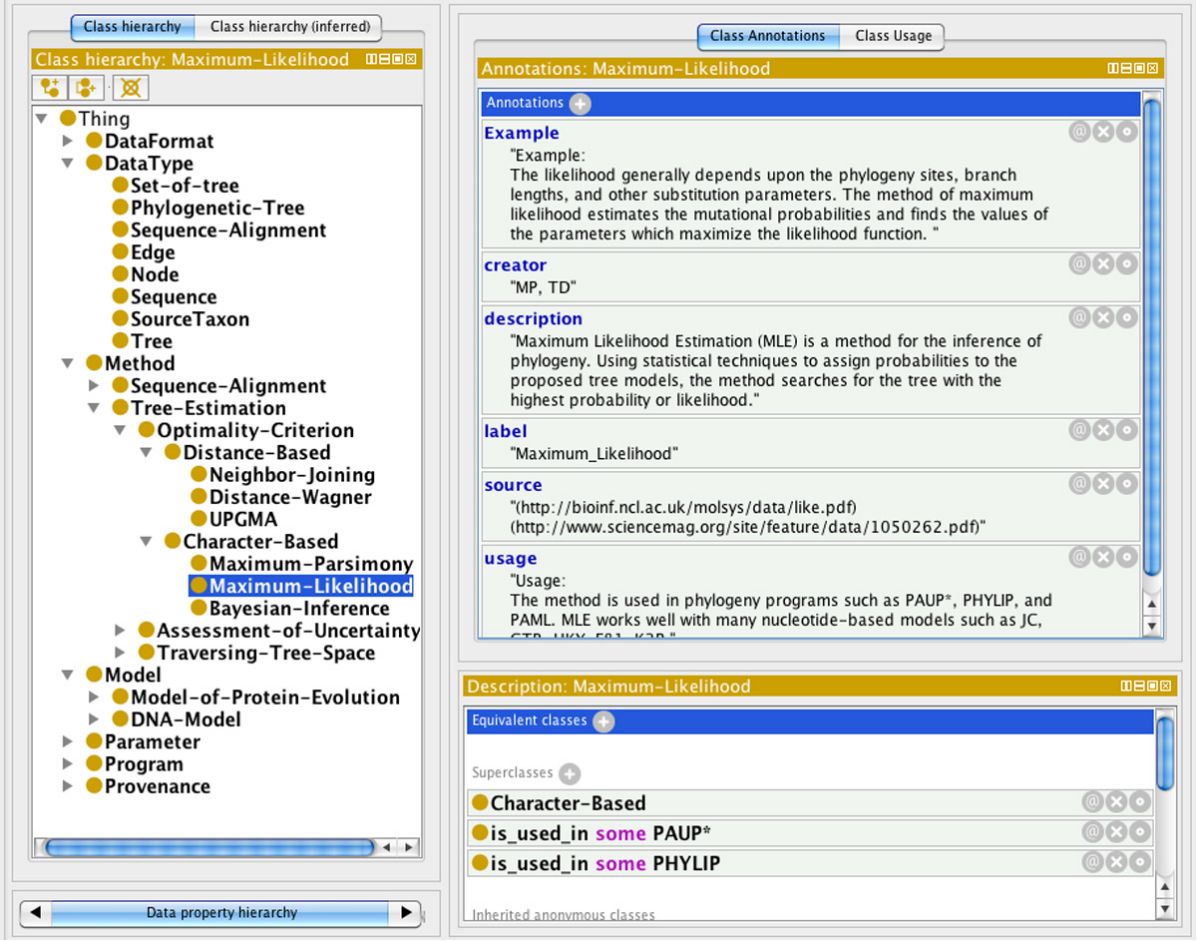
**PhylOnt implemented with Protégé**. The *PhylOnt *ontology as represented in Protege. The ontology includes descriptions of classes, definitions, properties, metadata, usage of classes with an example and relations among them.

In developing *PhylOnt *we worked closely with phylogeneticists and computer scientists to iteratively validate the ontology based on community feedback. As shown in Figure 1, development of *PhylOnt *started with data collection and organization of concepts in relational diagrams. Concept maps drawn from the primary literature included descriptions, properties, metadata, usage of concepts and relations between them. Subsequently, the version of *PhylOnt *presented here was developed in Protege 4.1.0 (Figure 2), which supports the Web Ontology Language (OWL). *PhylOnt *is accessible at NCBO through BioPortal [21]. A phylogentics domain specific extension of the Kino annotation package [12,13] was used in *PhylAnt *platform to facilitate annotation and faceted search over the annotated resources including scientific literature and NeXML format data files.

#### Data collection

Data resources in phylogenetic studies can be classified into primary and metadata categories. Primary data exist as published data files, literature with text, images, Excel files, and other supplementary materials. Primary data can also refer to methods, models, programs and even parameters used in applications and web services. Metadata includes information such as when and where the primary data were created. This information plays a very important role in enabling reusability.

To perform data extraction, a well-framed approach was required to identify and capture steps in phylogenetic workflows described in published phylogenetic studies [17]. We used *PhyloWays *[22], as a set of interpreted phyloinformatic workflows described in the primary phylogenetics literature. We identified all the information required to repeat the analysis presented in the *PhyloWays *papers, including the phylogeny estimation programs used in each paper, methods of analysis, evolutionary models and provenance information. These descriptions paved the way for classification of concepts associated with phylogenetic data (including provenance information), phylogenetic workflows, and the results of phylogenetic analysis.

Based on discussions with domain experts, literature reviews and the data in *PhyloWays *we then created concept maps describing methods of phylogenetic analysis, evolutionary models used in applications of these methods, and widely used phylogenetic software.

#### Methods of phylogenetic analysis

**Figure 3:**
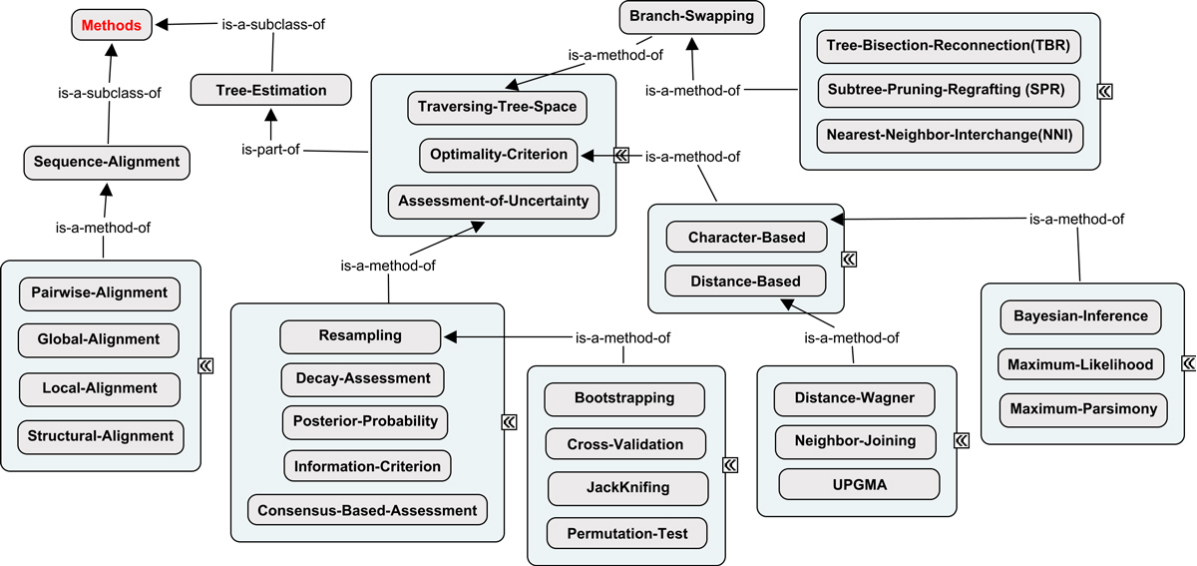
**Methods in phylogenetic studies**. A hierarchy of concepts used to describe methods commonly used in phylogenetic studies.

Phylogenetic methods vary considerably in approaches for assessing alternative hypotheses (i.e. trees), traversing through the complex universe of alternative hypotheses (i.e. tree and model parameter landscapes) and characterizing the degree of support for an optimized solution. As shown in Figure 3, a hierarchical classification of optimality criteria, search algorithms and uncertainty assessment concepts is implemented in *PhylOnt*. For example, tree inference methods based on maximum parsimony, maximum likelihood or Bayesian statistics rely on the analysis of a homologized character state matrix, whereas UPGMA, neighbor joining and distance-Wagner are based on sets of pairwise distances that may be estimated from a character state matrix or computed in some other way.

The universe of possible trees is extremely complex and identifying the optimal tree in this tree landscape is an NP-hard computational problem. Therefore, there is a variety of heuristic approaches for traversing the tree space in search of the optimal tree. Most maximum parsimony and maximum likelihood analysis methods build an initial tree and then iteratively test for improvement by rearranging the tree topology using branch-swapping algorithms such as nearest neighbor interchange (NNI), subtree pruning and regrafting (SPR), tree bisection and reconnection (TBR), or combinations thereof. Bayesian inference methods also include a branch-swapping process within a Markov Chain Monte Carlo (MCMC) strategy for sampling tree space.

Assessment of support for a phylogenetic inference is key in deciding whether an optimized solution is acceptable [23]. Bayesian inference methods provide posterior probabilities for the relationships conveyed in a phylogenetic tree, whereas other methods typically use bootstrap or jackknife resampling to assess the degree of support for hypothesized relationships. Resampling approaches can be combined with MCMC sampling in Bayesian analyses and the process of randomly resampling the original data matrix typically reduces posterior probabilities relative to those reported for MCMC searches without resampling [4].

#### Models in phylogenetic analysis

**Figure 4:**
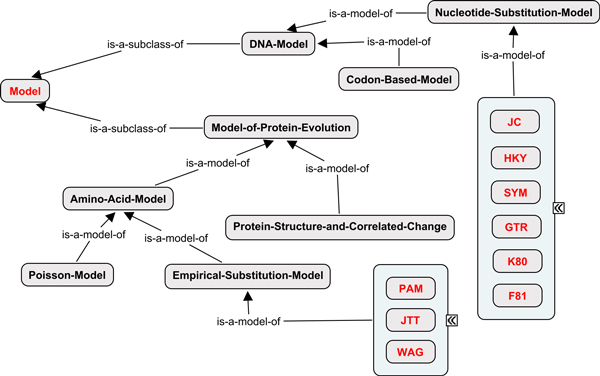
**Models in phylogenetic studies**. A hierarchy of concepts used to describep evolutionary models most commonly used in phylogenetic studies.

All phylogenetic analyses are performed with an explicit or implicit model of character evolution. Maximum likelihood, Bayesian inference and most distance-based methods rely on nucleotide or amino acid substitution models. Branch lengths for phylogenetic trees often represent time or evolutionary change. Correct interpretation of branch lengths requires an understanding of the models used to estimate time or evolutionary change. Separate substitution models are used for analyses of DNA and protein sequence alignments. Nucleotide substitution models include JC69, K80, HKY85, SYM, F81, and GTR [24,25]. Commonly used amino acid substitution models include PAM [26], JTT [27] and WAG [28]. Gene sequences typically include conserved domains and less conserved regions. The resulting among-site variation in substitution rates is often modeled in phylogenetic analysis of either nucleotide or amino acid alignments using a discrete approximation of the gamma distribution [29], a fraction of invariant sites [30], or a combination thereof. Both of these forms of rate variation can be layered upon the nucleotide and amino acid substitution models described above. Figure 4 shows a hierarchy of concepts used to describe evolutionary models most commonly used in phylogenetic studies.

Phylogenetic methods and the models they use are constantly changing as the phylogenetics community works to make more accurate and precise inferences about relationships and evolutionary processes. Therefore *PhylOnt *is necessarily incomplete, but easily extended to include additional models.

#### Programs in phylogenetic analysis

At time of writing, there are approximately 400 phylogeny packages and more than 50 free web servers for phylogenetic analysis [17]. *PhylOnt *currently identifies the most commonly used phylogenetic inference programs such as MrBayes [31], and PAUP* [32]. Programs can be categorized based on the methods they use. For example, PAUP* can be used to perform most major methods of analysis such as maximum parsimony and maximum likelihood. For more details about the programs, such as description for each and relation between programs, models and methods readers are referred to the *PhylOnt *project page on BioPortal [21].

### PhylAnt, a platform for semantic annotation, indexing and searching of phylogenetic resources

Semantic annotation maps target data resources to concepts in ontologies. In the process of annotation, extra information is added to the resource to connect it to its corresponding concept(s) in the ontology. *PhylAnt *offers a semi-automatic approach for such annotation of phylogenetic resources with the help of a suite of tools called *Kino-Phylo*. The complete suite of tools and instructions can be found at [33].

#### Annotating phylogenetic documents with Kino-Phylo

Kino for phylogenetics, also known as *Kino-Phylo *[13,17] is built on top of the Kino platform [12,33]. It is an integrated suite of tools that enables scientists to annotate phylogeny related documents in the *PhylAnt *platform. *Kino-Phylo *can annotate documents by accessing *PhylOnt *and other NCBO ontologies, via the NCBO Web API.

*Kino-Phylo *presents a comprehensive architecture for annotating and indexing phylogenetic oriented resources that should be of great use for the phylogenetic community. This system includes two main components, a browser-based annotation front-end, integrated with NCBO and an annotation-aware backend index to provides faceted search capabilities. It is designed around a basic workflow consisting of three steps, annotation, indexing, and searching[17,12]:

1. Annotation: In the annotation step, users provide annotations via a browser plug-in. After the annotations are added, the augmented document can be directly submitted to the indexing engine.

2. Indexing: Indexing is performed using Apache SOLR. It can be installed as an independent application and exposes multiple interfaces for client programs. SOLR provides the isolation for the index as well as support for faceting. Note that the SOLR interfaces are not directly exposed. They are wrapped by the *Kino-Phylo *submission API, described later in this paper. The annotation-aware back-end index is exposed via a RESTful API. It is designed such that the browser plug-in can directly submit the annotated web pages to the indexing engine.

3. Search: The search is performed via a Web interface. It presents the notions of a typical search engine and additionally gives the ability to filter the results via the facets. The current UI is built upon the JSON based Kino search API, which can be used to integrate other tools as well.

#### Browser plug-in for phylogenetic annotation

**Figure 5:**
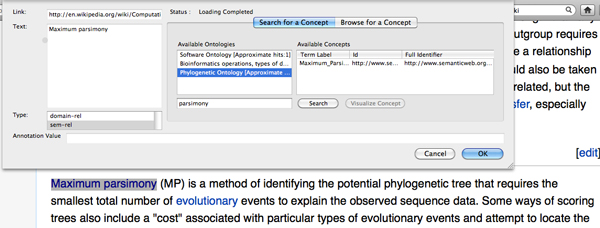
**Annotation of phylogeny literature with Kino-Phylo Tools**. The *Kino-Phylo *for the literature annotation plug-in shows how papers may be annotated. When the user highlights and right clicks in a term or phrase for annotation, the browser's context menu includes the annotation as a phylogenetical concept menu item. Selecting this menu item brings the annotations window where the highlighted term is searched using the NCBO RESTful API and a detailed view of the accessible ontological terms is shown to the user to choose the term from ontology and assign for annotation.

To use the browser plug-in, the user opens a topical web document in her browser and highlights words and phrases of interest. The plug-in provides hints on matching concepts fetched from NCBO. The user can also opt to browse for a concept in any ontology in NCBO (Figure 5). Once the annotations are added, the user can submit the annotations to a predefined *Kino-Phylo *instance (configured via the plug-in configuration page), by selecting the "publish annotations" menu item.

The plug-in modifies the HTML source of the document and embeds annotations using the SA-REST specification[12]. At submission, the augmented document tree in the browser is serialized and submitted as XML to the back end index via the document submission API (See next section).

#### Kino-Phylo index and search manager

The *Kino-Phylo *index manager is based on the Java JSP/Servlets technology and includes two major components, Document Submission API and Search API. The submission API acts as the receiver for the submitted documents. After receiving a document via the Document Submission API, the document will be filtered for embedded annotations and indexed. The index runs full-text indexing and special indexing for the filtered-out concepts. Additionally, the indexing process extracts extra information (such as synonyms) via NCBO and inserts this information in the index as well.

The *Kino-Phylo *search API includes a selection window that helps users to filter search results. For example, a user can search for parsimony as a concept or as a word. Once she finds a set of documents, they can be further filtered by co-locating concepts. For example, she can filter out the documents that have annotations on parsimony only across the documents that contain parsimony as an annotation for the methods used in phylogeny study. The User Interface includes an intuitive facet selection tool that helps the user to filter the results.

#### Annotation of NeXML files with Kino-Phylo

Vos, et al. [11] proposed NeXML as an exchange standard for representing phylogenetic data, inspired by the commonly used NEXUS format [34], but more robust and easier to process. XML formats such as NeXML play an essential role in promoting the accessibility and reuse of data on the web. Using this technology can simplify and improve robustness in the processing of rich phylogenetic data and enable their reuse.

**Figure 6:**
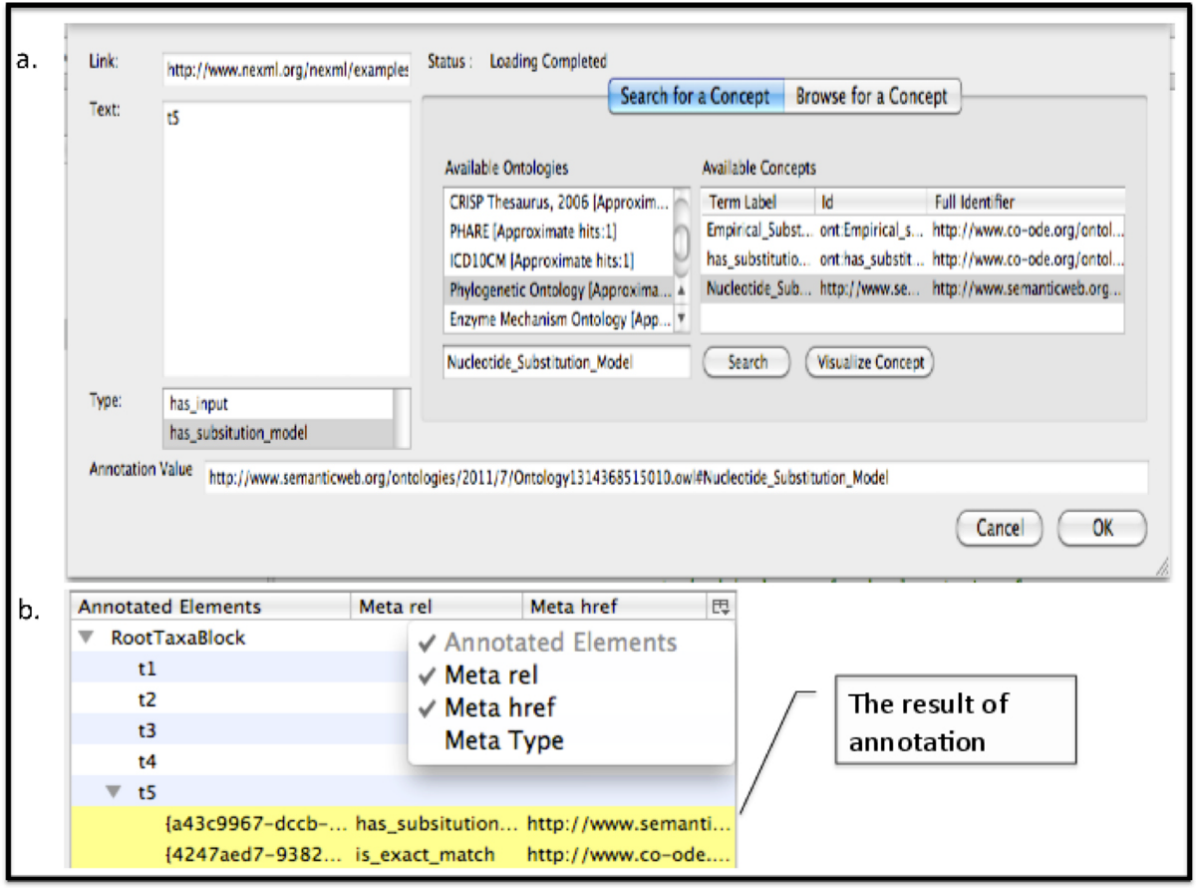
**Annotation of NeXML file with Kino-Phylo Tools**. The *Kino-Phylo *user interface for NeXML annotation plug-in shows how users can identify concepts from any NCBO ontology including *PhylOnt *using exact or approximate searches to annotate selected element in a NeXML file with the triples from ontology.

Annotations in NeXML are expressed using recursively nested "meta" elements that conform to RDFa syntax. The annotations thus form triples of subject, predicate, and object, where the subject is either a fundamental data resource from the NeXML document such as a tree, character state matrix, or taxon; or, transitively, the object of another triple. Instead of trying to provide vocabulary for all metadata types within the NeXML standard, users can thus use vocabularies or ontologies in common usage in the phyloinformatics community to annotate NeXML fles. To demonstrate this facility, we annotated NeXML documents using *Kino-Phylo*. With this approach, users can identify concepts from any NCBO ontology using exact or approximate searches to annotate selected element in a NeXML file (Figure 6). Users can then annotate a NeXML element to the desired triple, so that a statement can be made such as (subject NeXML element) "tree" (predicate) *has − substitution − model *(object) *nucleotide − substitution − model*.

## Results and evaluations

*PhylOnt *is publicly available. As shown in Figure 2, this ontology includes descriptions of classes, definitions, properties, metadata and usage of classes with an example for each one and relations between them. With the help of NCBO researchers, *PhylOnt *has been deployed on BioPortal, a web-based portal designed and hosted by NCBO to enable accessibility to biological knowledge on the Semantic Web. In the comparison of PhlOnt at [17] In addition, we introduced and implemented *PhylAnt *platform for annotating, indexing and searching phylogenetic resources such as scientific articles and NeXML files.

### Evaluation

Ontology evaluation is needed to guarantee that what has been built meets application requirements. There are different approaches for ontology evaluation, such as metric-based and application-based [35]. In the following sections we present results from both approaches.

#### Metric-based approach

Metric-based evaluations scan through an ontology to gather different types of statistical criteria about structural knowledge represented in an ontology. We used schema metrics [35], which evaluate ontology design and its potential for rich knowledge representation. Table 1 shows the results of these evaluations. "Relationship richness" reflects the diversity of the types of relations in the ontology, which is now higher in the comparison of our previous work [17] "Attribute richness" indicates both quality of ontology design and the amount of information pertaining to instance data. The results of the relationship richness assessment show that 74 percent of the connections between classes are "rich" relationships compared to all of the possible connections.

**Table 1:** Metric-Based Approach for Ontology Evaluation

Metric name	**Metric formula**1	Metric value
Relationship Richness	$RR=\frac{\left|P\right|}{\left|H|\;+\;|P\right|}$	0.74
Attribute Richness	$AR=\frac{\left|T\right|}{\left|C\right|}$	0.30

^1^|*P*|: Number of non-inheritance relationships, *|C|*:Number of classes, *|T |*:Number of attributes

We also compared the *PhylOnt *Ontology with the Comparative Data Analysis Ontology(CDAO) [36,19] and the Embrace Data And Methods(EDAM) [20] ontology for these metrics. Table 2 shows the result of this comparison. *PhylOnt *includes deeper resolution of concepts related to phylogenetic inference methods, substitution models, tree estimation programs and provenance.

**Table 2:** Numerical Comparison of Ontologies EDAM, CDAO, PhylOnt

Parameters	EDAM	CDAO	PhylOnt
Number of classes	2746	143	147
Phylogeny analysis terms	26	129	138
Phylogeny methods	9	8	41
Substitution models	2	NA	31
Phylogeny programs	8	14	33
Provenance	NA	NA	21
Phylogeny data and Types	5	NA	12

#### Annotation-based approach

A fundamental driving principle for the development of ontologies is their utility for data annotation and management. Therefore, as we developed *PhylOnt*, we evaluated it by annotating resources in phylogenetic documents using *Kino-Phylo *tools.

Collaborating domain experts selected exemplar publications that we used to investigate which concepts are missing in *PhylOnt *by trying to annotate the exemplars with it. The rationale is that we could determine the quality of *PhylOnt *by counting the relevant concepts encountered in a paper that are not present in *PhylOnt*, but are present in other relevant ontologies. This approach is used to compute Precision, Recall, and F-measure [37]. Suppose that *C*_{*P*∩*O*} _is the set of concepts from the papers that have been annotated using *PhylOnt*. Then Precision and Recall can be calculated by the following equations:

(1) $$Precision=\frac{\left|C_{\left\{P\cap O\right\}}\right|}{\left|C_{P}\right|}$$

(2) $$Recall=\frac{\left|C_{\left\{P\cap O\right\}}\right|}{\left|C_{O}\right|}$$

*C_P _*and *C_O _*refer to the concepts of the paper and concepts in ontology respectively. The F-measure is the harmonic mean of precision and recall and it is calculated as:

(3) $$F-measure=\frac{2\times Precision\times Recall}{Precision+Recall}$$

**Figure 7:**
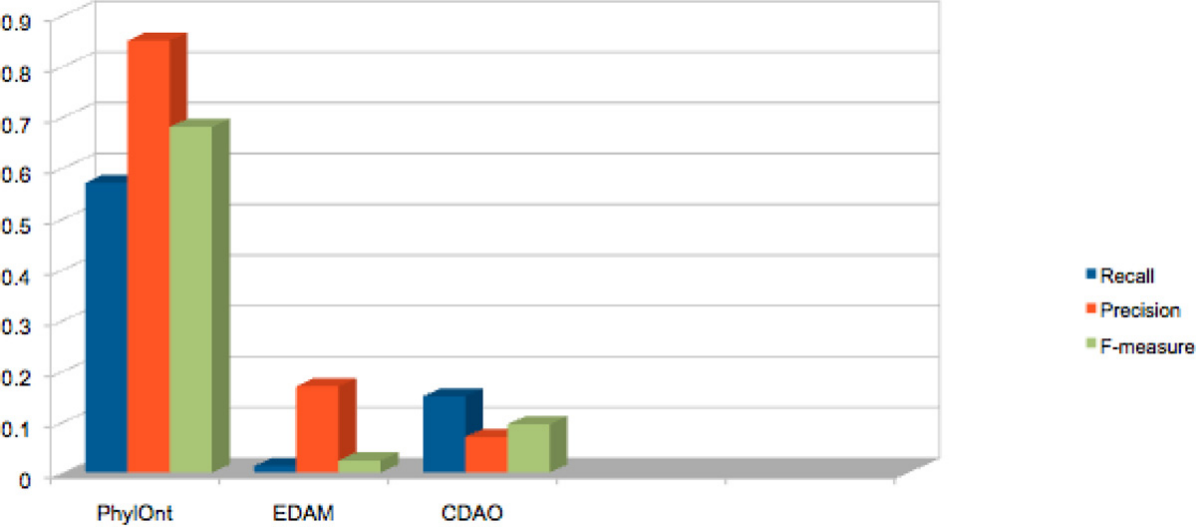
**Annotation-based approach for ontology evaluation**. Precision, recall and f-mature calculated for PhyLOnt in the comparison of EDAM and CDAO Ontology. We increased the number of concepts in PhylOnt in vers8 of ontology [21]. After annotation the expert selected papers with ontology, the precision is higher than our previous published results [17].

For this experiment, we annotated selected papers by experts using *PhylOnt*, EDAM and CDAO. We increased the number of concepts in PhylOnt in vers8 as of Dec 2012 after getting feedback from community. After annotation the expert selected papers with ontologies, the precision is higher than our previous published results [17]. As it is shown in Table 3. the precision score of *PhylOnt *indicates that 85% of the phylogenetics concepts in the papers are covered by the ontology, while 57% of all concepts in the ontology are recalled in the selected papers. In combination, these scores were used to estimate an F-measure for *PhylOnt *that is higher than that of CDAO and EDAM (Figure 7).

**Table 3:** Annotation-based approach for ontology evaluation

Ontology	Precision	Recall	F-measure
PhylOnt	0.85	0.57	0.68
EDAM	0.17	0.013	0.024
CDAO	0.07	0.15	0.095

## Discussion

A big challenge in phylogenetic studies is the complexity of data being used in phylogenetic reconstruction and the diversity of analysis methods. Some of the barriers to reuse of this data are incomplete and non-tractable provenance data; insufficient method descriptions to reproduce the results; and the lack of semantic annotations of resources. Our focus in this study was on formally characterizing phylogenetic resources and identifying the relationships among key concepts. To the best of our knowledge and the feedback from the phylogenetics community [13], *PhylOnt *is the first ontology specifically created for phylogenetic analysis operations and related metadata.

As of March 2013, the 8th version of *PhylOnt *has been submitted to NCBO. Our results show that *PhylOnt *is a rich ontology for the concepts in phylogeny applications compared to putative alternatives such as EDAM and CDAO [17]. Note, however, that the EDAM ontology is much more broadly scoped to the entire bioinformatics domain, whereas CDAO is scoped to defining the relationships among fundamental data concepts (e.g. nodes, trees, character state matrices), not methods of phylogenetic analysis or provenance metadata. As real-world use cases of richly annotated phylogenetic data develop it is likely that these three artefacts will therefore be complementary rather than in competition.

We introduced the *PhylAnt *platform, which enables semantic annotation of phylogenetic resources. Annotating phylogenetic documents using ontologies is the foundation for the use of other semantic technologies in this domain and it is a preliminary step to semantic search, information retrieval, and heterogeneous data integration that can support phylogenetic workflows. These annotations have a variety of uses, ranging from extended search capabilities to advanced data mining. Annotated documents are indexed using a faceted indexing and search engine that provides fine-grained search capabilities.

*PhylOnt *does not currently cover all concepts included in phylogenetic analyses, but rather forms a foundation for an extensible ontology that will grow as researchers develop and apply new analysis methods. Further, the ontology does not currently include all method or model specific parameter definitions. Again, these can be added to the ontology as needs are defined by the phyloinformatics community.

## Conclusion

The research presented in this manuscript is aimed at applying semantic web technologies to phyloinformatics. We addressed these objectives from both a phylogenetics and a computer science perspective. From the phylogenetics community perspectives, reusability and the ability to search for phylogenetic information are improved with the help of semantic web technology. From a computer science perspective, semi-automatic annotation of different resources with the concepts defined in *PhylOnt*, indexing and searching through resources will facilitate interoperability among phylogenetic resources. These advances allow researchers to access, explore and reuse the resources and products of phylogenetic studies.

## Competing interests

The authors declare that they have no competing interests.

## Authors' contributions

MP conceived of the study, ontology development, annotation and drafted the manuscript. AS participated in semantic approaches for data reuse. AR participated in annotation and searching framework for both literature and NeXML documents. RV participated in NeXML annotation process. JL participated in study design in phyloinformatics, ontology development, annotation, validation and helped to draft the manuscript. All authors read and approved the final manuscript.

## Declarations

The publication costs for this article were funded by Jim Leebens-Mack.

This article has been published as part of *BMC Medical Genomics *Volume 6 Supplement 3, 2013: Selected articles from the IEEE International Conference on Bioinformatics and Biomedicine 2012: Medical Genomics. The full contents of the supplement are available online at http://www.biomedcentral.com/bmcmedgenomics/supplements/6/S3.
